# Testing a Benchtop Wet-Milling Method for Preparing Nanoparticles and Suspensions as Hospital Formulations

**DOI:** 10.3390/pharmaceutics13040482

**Published:** 2021-04-02

**Authors:** Yayoi Kawano, Yuichiro Shimizu, Takehisa Hanawa

**Affiliations:** Faculty of Pharmaceutical Sciences, Tokyo University of Science, 2641, Yamazaki, Noda, Chiba 278-8510, Japan; y.kawano@rs.tus.ac.jp (Y.K.); shimizu.y.1126@gmail.com (Y.S.)

**Keywords:** hospital formulation, cefditoren pivoxil, ternary ground mixture, dispersibility, nanoparticle, wet-milling technique

## Abstract

In clinical practice, for elderly or pediatric patients who have difficulty swallowing, solid dosage forms such as tablets or capsules are crushed or unsealed, prepared as powder forms, and often administered as suspensions. However, because their dispersibility is poor, aggregation or precipitation occurs readily. Once precipitation and deposition happen, redispersion is difficult, which can limit patient and caretaker drug adherence. In this study, we attempted to prepare nanoparticles as a hospital formulation by a benchtop wet-milling method to obtain a suspension with high dispersibility. This is the first study to apply the wet-milling method to prepare the hospital formulation. We chose cefditoren pivoxil (CDTR-PI) as an experimental active pharmaceutical ingredient. CDTR-PI crystals were physically mixed with various water-soluble polymers such as polyvinylpyrrolidone, polyethylene oxide, hydroxypropyl cellulose, or hypromellose and wet-milled with a surface-active agent (sodium lauryl sulfate) under different conditions. The mean particle diameter of most of the samples was less than 200 nm. In FTIR spectra of ground samples, peak shifts suggesting inter- or intramolecular interactions between CDTR-PI and the other additive agents were not observed. Besides, the nanoparticle suspension had favorable dispersibility, as determined using a dispersion stability analyzer. Providing a suspension with high dispersibility makes dispense with the resuspension, the patient’s medication adherence would improve. These results show that suspended liquid formulations of active pharmaceutical ingredients could be obtained by the simple wet-milling method as hospital formulations.

## 1. Introduction

Recently, the elderly population has been increasing worldwide, and many elderly patients have dysphagia. Furthermore, young pediatric patients also struggle to swallow solid dosages as tablets, capsules, and even fine granules. Better hospital formulations have been required for these patients.

A variety of formulations have been developed for many medicines. However, dosage forms such as tablets are sometimes crushed by pharmacists to administer for children, because there is no optimal dosage for children in Japan. Additionally, in a realistic medical setting, such as a hospital, home medical care, or palliative care, various medicines are available to respond to patient needs; there are often cases where the existing medicine cannot treat each patient. To solve these problems, various hospital formulations have been developed and applied in Japan. Hospital formulations are defined as medically required formulations that are not commercially available, but prepared by a pharmacist in the hospital according to a medical doctor’s request and used entirely within that institution. Many hospital formulations are prepared by pharmacists when faced with specific medical needs and are supplied only to hospitals. 

In hospital formulations, the active substances from commercial products have been often used as active pharmaceutical ingredients (APIs). However, more than 40% of pharmaceutical compounds have extremely poor water solubility [1,2]. To develop a method for preparing the hospital formulation using such water-insoluble APIs, we focused on cefditoren pivoxil (CDTR-PI) as a model API in this study. CDTR-PI is a third-generation oral cephalosporin with a broad spectrum of activity against pathogens, including Gram-positive and Gram-negative bacteria, and is stable against hydrolysis by many common β-lactamases. CDTR-PI is approved for acute bronchitis, pneumonia, acute maxillary sinusitis, acute pharyngitis, and tonsillitis, although indications may differ among countries [3]. CDTR-PI is synthesized as a prodrug of pivoxil ester to increase its bioavailability and is in Class IV of the Biopharmaceutics Classification System due to its poor water solubility (<0.1 mg/mL) and permeability [4,5].

In the US, Asia, and Europe, CDTR-PI is administered as tablets or fine granules. Fine granules were containing 100 mg/g as CDTR-PI is mainly prescribed for children in Japan, South Korea, and Turkey. However, when elderly patients with dysphagia take tablets or fine granules, they risk suffocation and aspiration pneumonia, leading to non-adherence with the dosage. To overcome these problems, a liquid dosage form such as a syrup or oral suspension is desirable. Generally, liquid dosage forms are classified as monophasic or polyphasic. Thus, dispersibility is an essential property in formulating liquid dosage forms. Many solubilization techniques have been developed to improve APIs’ dispersibility, such as self-emulsification, solid dispersions prepared by coprecipitation, spray-drying, freeze-drying hot-melt extrusion, and complex formation with water-soluble excipients. Those methods can often result in an amorphization of drug crystals; an amorphous state is unstable, the crystallization of the drug is followed by a decrease in concentration [6]. 

This past decade, particle size reduction including the use of attrition-milled nanocrystalline forms [7]. Among these, the wet-milling technique using a water-soluble polymer and a surfactant is known as “top–down production methods”, to be a beneficial and easy method that can reduce particle diameter [8,9,10]. Primarily, nanoparticle (NP) has been focused as drug carrier based on their unique features such as their large surface and their ability to adsorb or carrying various APIs [11].

We focused on nano-suspension as a liquid dosage form prepared by the wet-milling technique from these points of view. In general, nano-suspension has better dispersion stability and membrane permeability than micro-suspension, because the particle size can be reduced, then, the sedimentation of the particles can be minimized. For this reason, we considered the preparation of nano-suspension for the hospital formulation, which has good dispersion stability.

The API is ground in a dispersion containing a water-soluble polymer and a surfactant using zirconia beads [8,9,10,12]. The particles can be reduced to 100–200 nm and dispersed in suspension by wet-milling [13]; some commercial formulations have been developed using this technology [14]. However, the grinding techniques mentioned above require special facilities and equipment. On the other hand, some benchtop equipment is known, such as a low energy ball mill and a micro-beads mill, used widely on lab-scale [8,9]. However, since these are still expensive and special for some hospital pharmacies when the technique is applied for the hospital formulation, usability and/or cost of instruments should be considered. As for the simple method for preparing a hospital formulation, we have reported the preparation of nanoparticles (NP) with hydroxypropyl cellulose (HPC; HPC-L, HPC-SL, and HPC-SSL) and sodium lauryl sulfate (SLS) using the wet-milling method to improve solubility and dispersibility, which was used a propeller-type stirrer and zirconia beads. We found the optimal time and the diameter of the beads for grinding [15]. Then, we selected the blade turbine impeller mixer, which is widely used and provides both radial and axial flow; additionally, it generates higher shear levels for mixing [15].

In this study, to prepare CDTR-PI NPs with high dispersibility as a hospital formulation, CDTR-PI was wet-milled with SLS and various grades of HPC (HPC-L, HPC-SL, and HPC-SSL), polyethylene oxide (PEO), hypromellose (HPMC), or polyvinylpyrrolidone (PVP) with zirconia beads. Firstly, we screened about optimal beads diameter and grinding time when polyethylene oxide (PEO) was used as water-soluble polymer and SLS. Secondly, HPCs were investigated as one of the water-soluble polymers using the same of our previous conditions. From these results, HPMC and PVP were studied under identical conditions as well. Furthermore, the nanosuspension’s physicochemical properties, such as particle size, zeta potential, solubility, crystallinity, molecular state, and dispersibility, were evaluated. The nanosuspension was assessed for possible use as a hospital formulation. No other studies of applying the wet-milling method to prepare the hospital formulations have been carried out, to the best of our knowledge.

## 2. Materials and Methods

### 2.1. Materials

PEO (PEO-1NF, molecular weight (MW): 150,000–400,000), HPC (HPC-L, HPC-SL, and HPC-SSL, MW: ~140,000, ~100,000, and ~40,000 respectively), HPMC (HPMC 60SH-50, MW: ~100,000), and PVP (PVP K-30, MW: ~40,000) were used as water-soluble polymers. PEO-1NF was provided by Sumitomo Seika Chemicals Co., Ltd. (Osaka, Japan). HPC-L, HPC-SL, and HPC-SSL were provided by Nippon Soda Co., Ltd. (Tokyo, Japan). PVP K-30 and HPMC were purchased from Nacalai Tesque, Inc. (Kyoto, Japan) and Shin-Etsu Chemical Co., Ltd. (Tokyo, Japan). Sodium lauryl sulfate (SLS) was purchased from Wako Pure Chemical Industries, Ltd. (Osaka, Japan). Purified water was used as the dispersion media. CDTR-PI was provided by Meiji Seika Pharma Co., Ltd. (Tokyo, Japan) (Supplementary Figure S1). All other chemicals and solvents were of analytical reagent grade.

### 2.2. Preparation of NPs

**Figure 1:**
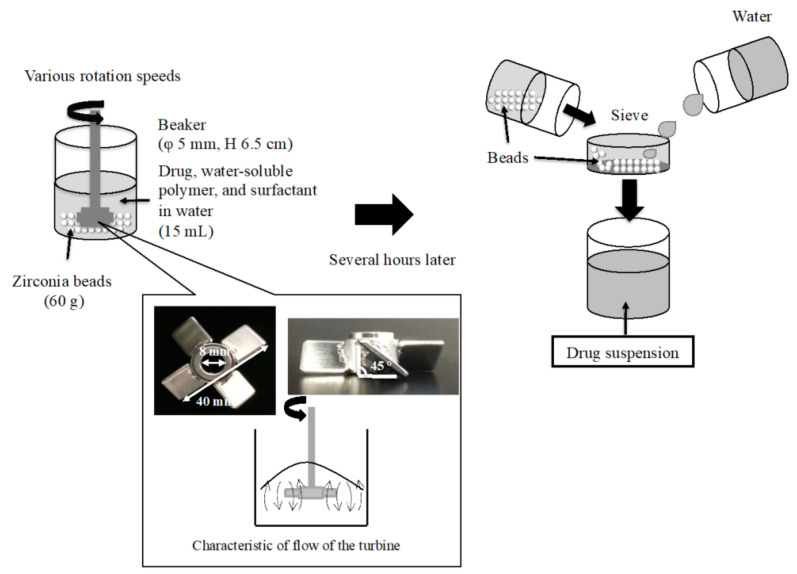
Schematic view of the wet-milling method using a propeller-type stirrer.

In this study, we tested a benchtop wet-milling method as one of the top–down production methods with reference to Niwa’s method [7]. A propeller-type stirrer (Three-One Motor, Shinto Scientific Co., Ltd., Tokyo, Japan) was used as the grinder. The diameter of the turbine blade impeller mixer was 40 mm, and the angle of the turbine blade was 45° (Tokyo Rikakikai Co., Ltd., Tokyo, Japan), which is called “mixed flow type” (Figure 1). Then, the characteristic of the flow of the turbine is shown in Figure 1. The angled turbine requires less power and is effective for solid–liquid agitation of materials with large specific gravity. Zirconia beads with various diameters (0.3, 0.5, and 1.0 mm) were purchased from Nikkato Co., Ltd. (Osaka, Japan) and used as the milling media. Figure 1 shows a schematic diagram of the wet-milling method using the propeller-type stirrer. The ternary mixtures consist of CDTR-PI, water-soluble polymer, and SLS were prepared as follows: briefly, CDTR-PI (0.6 g) and approximately 60 g of zirconia beads with various bead diameters (0.3 mm) were added to the aqueous dispersion medium (15 mL) containing 0.1% (*w/v*) SLS and different concentrations (0.25, 0.50 or 1.0% (*w/v*)) of PEO, HPCs, HPMC, or PVP. Firstly, the 0.5% PEO solution was stirred at various speeds (500, 710, or 900 rpm) for 3–24 h continuously using the propeller-type stirrer at 25 °C to be screened the optimal condition in this method. Afterward, other solutions were stirred at 500 rpm for 24 h continuously at 25 °C. After grinding, the beads were separated using a sieve and washed with purified water. The obtained filtrate was considered a suspension of CDTR-PI. The total solids’ concentration, including CDTR-PI, polymers, and SLS in the suspension, was adjusted to 1% *w/v* (Figure 1). 

### 2.3. Physicochemical Properties of NPs in Suspension

#### 2.3.1. Observation of NPs with Scanning Electron Microscopy (SEM)

As pretreatment before observation, some processes were performed as follows: 100 μL of suspension were added into 900 μL of purified water, and then, the sample was stirred and centrifuged at 15,000 rpm (21,652.65× *g*) for 3 min. Afterward, the supernatant was removed, and 1 mL of purified water was added to the precipitate, which was then suspended with an ultrasonic wave for 1 min; then, the obtained suspension was re-centrifuged at 15,000 rpm (21,652.65× *g*) for 3 min. After the centrifugation, the supernatant was removed, and 50 mL of purified water was added to the precipitate. The suspension was exposed to an ultrasonic wave for one minute and was dried with a dryer. After the pretreatment, obtained samples were fixed to the sample stage with gold using an ion coater and observed using SEM (JSM-6390LA, Jeol Ltd., Tokyo, Japan) at an acceleration voltage of 20 kV.

#### 2.3.2. Measurement of Particle Size of NPs in Suspension

Particle size was measured using a dynamic light scattering method with a zeta potential dynamic light scattering analyzer (ELSZ-2000ZS, Otsuka Electronics Co., Ltd., Osaka, Japan). Before measurement, 10 μL of the sample solution was diluted with added ultrapure water until the glass cell’s top. The mean diameter was calculated from a cumulant fitting analysis. The average was obtained using three samples. The obtained data of particle size were evaluated statistical analysis performed by Turkey–Kramer test. 

#### 2.3.3. Measurement of Zeta Potential of NPs in Suspension

The zeta potential of NPs in the suspension with and without SLS was measured using a zeta potential dynamic light scattering analyzer (ELSZ-2000ZS, Otsuka Electronics). Then, 50 μL of the sample solution was diluted with 3 mL of physiological saline for analysis. The charged particles’ electrophoretic movement was determined from the scattered light’s Doppler shift under an applied electric field. The zeta potentials were calculated using the Smoluchowski equation (Equation (1)).

(1) $$\mathrm{Zeta}\;\mathrm{potential}\;\left(\zeta \right)=\frac{4\Pi \eta U}{\varepsilon }$$

*η*: solution viscosity, *ε* the solvent dielectric permittivity, and *U*: the electrophoretic mobility mean zeta potentials are shown as the mean ± S.D. of three measurements. 

#### 2.3.4. Evaluation of Molecular Interactions in Suspension

Molecular interactions in the nanosuspension were evaluated using attenuated total reflection Fourier-transform infrared (FTIR) spectroscopy (FTIR spectrometer, PerkinElmer Co., Ltd., Waltham, MA, USA). The thickness of the sample was 1.0 mm. The suspension was ultra-centrifuged (Himac CP80MX) at 30,000 rpm (86,610.6× *g*) for 60 min to separate the solid and aqueous phases. The solid phase underwent FTIR spectroscopy in a scanning range of 500 to 4000 cm^−1^. 

#### 2.3.5. Measurement of Viscosity of Suspensions

The sample solutions’ viscosity was measured using a viscometer (LV DV2T, cone rotor: CPA-40Z; shear rate: 7.50 N∙s^−1^; the amount of sample solution: 0.5 mL; Brookfield Engineering, MA, USA). The temperature of the flow jacket for temperature control was kept constant at 25 ± 1 °C. Viscosity is reported as the apparent viscosity observed at a defined rotary speed (50 rpm).

#### 2.3.6. Evaluation of Crystallinity of NPs in Suspension

The samples were centrifuged (Himac CP80MX, Hitachi Koki Co., Ltd., Tokyo, Japan) at 30,000 rpm (86,610.6× *g*) for 60 min to separate the solid phase. Powder X-ray diffraction (PXRD) was carried out on a RINT 2000 (Rigaku Co., Tokyo, Japan). Measurements of crystallinity of obtained solid phase were performed at 40 kV voltage, 40 mA current, scanning speed of 4°/min, Ni filter, and a radiation source of CuKα_1_. 

### 2.4. Evaluation of Dispersion Stability of the Suspension

Dispersion stability of the suspension was examined at room temperature (25 °C) using a Turbiscan MA 2000 dispersion optical analyzer (Formulaction, Toulouse, France). The calibration of the instrument was automatic then performed before each scan. Five milliliters of the suspension, which consisted of 1% (*w/v*) as a solid component, included CDTR-PI, HPC-SSL, and SLS was placed immediately in a cylindrical glass cell after grinding and analyzed using a near-infrared light beam (λ = 850 nm) which scans the sedimentation column. Two synchronous detectors detected the light transmitted through the product (at 0° from the incident beam) and backscattered by the product (at 135° from the incident beam). The spectra in Supplementary Figure S2 represent the typical transmittance and backscattering profile obtained by Turbiscan, then used by evaluating turbidity or dispersion stability in general. Low concentration samples can be assessed using the transmittance alone. However, high concentration samples use backscattering because these cannot transmit light. In this study, we also evaluated the dispersion stability of suspension using transmittance and backscattering profiles. The X-axis represents the distance from the bottom of the test tube (mm), and the Y-axis represents the backscattered light percentage (%). The detection head scanned every 24 h over 14 days at room temperature, the entire length of the sample while acquiring transmittance and backscattering data every 40 μm. Then, the sample tube had placed on the cell of the instrument for 14 days. 

### 2.5. Measurement of Solubility

CDTR-PI solubility in the suspension whose polymers are HPC-SSL 0.5% and PVP 0.5% was measured by HPLC (SSC-3461; Senshu Scientific Co., Ltd., Tokyo, Japan). The suspensions were filtered using a syringe equipped with a cellulose acetate filter (pore size 0.2 μm), and 40 μL of each filtrate was injected into a C18 column (PEGASIL ODS SP100 (φ 4.6 mm × 150 mm); Senshu Scientific Co., Ltd., Tokyo, Japan) at 25 °C. The flow rate was 1.0 mL/min for all separations, and the mobile phase consisted of the formic acid buffer, acetonitrile, and methanol at a volume ratio of 18:11:11. Sample concentrations were quantified at a wavelength of 230 nm.

## 3. Results and Discussion

### 3.1. Effect of Various Grinding Conditions on the Particle Size of NPs

**Figure 2:**
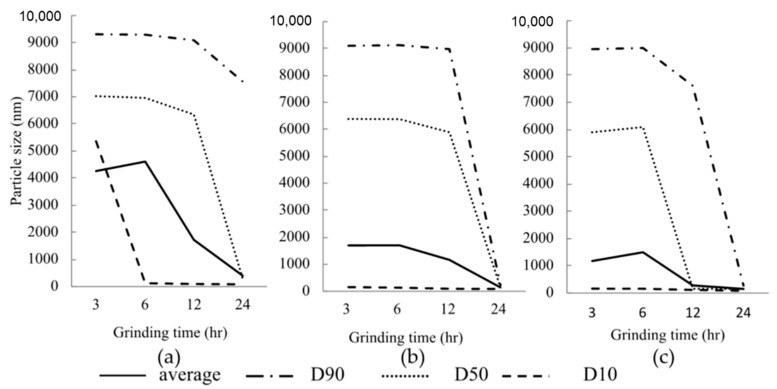
Effect of the diameter of zirconia beads and grinding time on the particle size of CDTR-PI when 0.5% *w/v* PEO solution was used as a water-soluble polymer with 0.1% *w/v* SLS. Diameters of zirconia beads: (**a**) 1.0 mm, (**b**) 0.5 mm, and (**c**) 0.3 mm.

Firstly, the effects of bead size and grinding time on the particle size reduction was investigated when PEO was used as a water-soluble polymer with SLS at 500 rpm of rotation speed. Figure 2 shows the particle size changes to the ternary mixtures’ grinding time consisting of CDTR-PI, 0.5% (*w/v*) PEO, and 0.1% (*w/v*) SLS ground with various bead sizes. The particle size of DTDTR-PI crystals showed 17,143.7 ± 1241.73 nm, although it is not shown in Figure 2. The particle size reduced with an increase in the grinding time then the temperature of suspension did not change during grinding in all samples. In all samples ground with 0.3, 0.5 or 1.0 mm zirconia beads, the median diameter (D_50_) reduced after 12 h. In particular, NPs were obtained in all grinding conditions after 24 h without changing temperature during grinding. As for the effect of grinding time and bead size on the particle size, as anticipated, a longer grinding time yields a finer particle size at a given bead size. Shin et al. demonstrated that the particle size reduction results from the grinding aid’s complicated dynamic interactions with the turbulent slurry during grinding [15].

Additionally, the number of contact points between beads might be an essential factor in the particle size reduction; because the small-size beads have a large surface area, they have many possible contact points. Our previous study showed this phenomenon using HPCs as a water-soluble polymer with the same conditions [16]. In this study, though only three types of beads were compared, and it is necessary to study the grinding effect of beads with more types of bead diameters in the future, it seemed that optimal grinding time and zirconia beads diameter are 24 h and 0.3 mm, respectively. 

**Figure 3:**
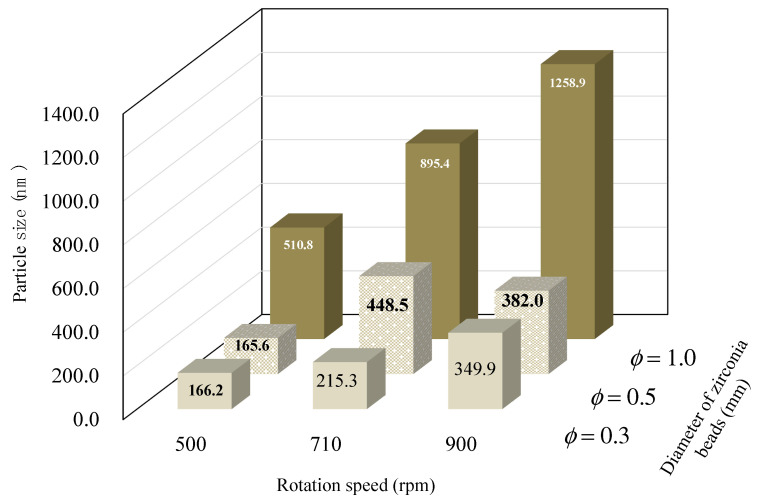
Effect of rotation speed and diameter of zirconia beads on the mean particle diameter of ground samples as used with 0.5% *w/v* PEO solution and 0.1% *w/v* SLS.

Figure 3 shows the effect of the propeller’s rotation speed on the particle size using 0.5% (*w/v*) PEO. The temperature change in suspension was monitored during the grinding; however, the temperature did not change during grinding even though rotation speed was higher in all samples. For any bead size, the sample with a rotation speed of 500 rpm showed the smallest particle size. Initially, we anticipated that the particle size would become smaller with increased rotation speed; contrary to the expectations, the particle size became bigger with increase in the rotation speed, we now consider that aggregation might result at higher rotation speeds. That means that standard procedure of wet-milling is carried out in the beaker, which is not the enclosed grinding chambers, they differ from the flow in other standard wet media milling devices with enclosed grinding chambers (e.g., wet stirred media mills) in that they generate free-surface flow. This seemed to create a low-stress, low-energy collision zone in the large volume of suspension at high rotational speeds (similar to a tumbling ball mill), which could result in slower fracture kinetics and coarser particles at the highest rotational speeds. 

As described above, there are many factors to obtain the optimal condition to formulate nanosuspension by wet-milling. Medarević et al. attempted to find out the optimal process parameters to formulate the nanosuspension with using a Box-Behnken experimental design [17]. In this study also, the use of closed chamber and the optimal conditions should be determined by the experimental design in future study.

Based on the results mentioned above, all subsequent NPs were prepared under the following conditions (bead size: 0.3 mm, rotation speed: 500 rpm, grinding time: 24 h).

### 3.2. Physicochemical Properties of NPs in Suspension

**Figure 4:**
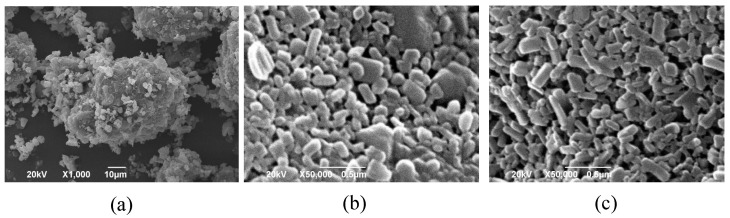
SEM images of bulk and ground cefditoren pivoxil particles. (**a**) CDTR-PI crystals, (**b**) HPC-SSL 0.5% + SLS 0.1%, and (**c**) PVP 0.5% + SLS 0.1%.

SEM images of the NPs in suspension and of CDTR-PI crystals are shown in Figure 4. The CDTR-PI crystals possessed irregular shapes with a particle size of greater than 10 μm (Figure 4a). In contrast, CDTR-PI particles grinding with HPC-SSL or PVP using the wet-milling method were column-shaped nanometer-sized particles (Figure 4b,c).

The particle sizes and zeta potentials of the suspensions prepared with HPMC, PVP, PEO, and the various HPCs are summarized in Table 1. As outlined in Table 1, most samples’ mean particle diameter was less than 200 nm, indicating that CDTR-PI crystals were ground to nanometer-size. Additionally, it was shown that each particle was ground effectively since each PDI was shown 0.13 to 0.22. The particle size tended to be lower at a low concentration of polymers. Comparing the particle size between samples prepared with various hydrophilic polymers showed different polymer concentration trends; however, all samples were obtained NPs. Notably, the particle size of NPs from HPC-SSL was 116.1–135.3 nm, and then they showed the smallest particle size in all polymers and each concentration.

Some studies have suggested that combining a water-soluble polymer and a surfactant may have synergistic stabilizing effects [18,19,20,21,22], such as inhibiting the aggregation of NPs. Pongpeerapat et al. 2008 demonstrated that in a ternary grinding system, the water-soluble polymer seems to form a macromolecular layer with SLS on the surface of APIs [18]. In fact, in this study, when CDTR-PI was wet-milled with polymer only, without adding SLS, the particle sizes were about 320–1280 nm (Table S1). Using wet-milling methods, the NPs preparation mechanism added water-soluble polymer as a stabilizer has been considered that polymers on their surface surround APIs particles. In this case, it was considered that CDTR-PI particles were surrounded by each polymer and SLS on their surface. HPC-SSL’s molecular weight is smaller than other polymers, so that the chain length is shorter than others. Then, it was considered that the overall particle size became smaller than others. Its phenomenon was observed in PVP, whose molecular weight is almost the same as HPC-SSL as well. As for the effects of the polymer concentrations on the grinding, the particle sizes decreased in the order of 1.0% > 0.25% > 0.5% in polymers except PVP suggesting the existence of an optimal concentration; however, the significant difference was not shown between 0.25% and 0.5% of concentration (Table S2). Then, it was suggested their viscosity led to the grinding effect. In particular, it was shown that HPCs have a good effect on grinding as a water-soluble polymer in this method. On the other hand, when PEO 1.0% (*w/v*) and HPMC were used as the water-soluble polymer in the present technique, their particle size showed around 200–400 nm, which was bigger than HPCs and PVP. It was suggested that polymers’ molecular weight and viscosity were related to the present technique’s grinding effect. It was considered that the shear force is not enough to cut polymer chain lengths additionally not to get enough rotation of flow in high viscosity solution because the shear force during grinding is low at 500 rpm. However, a decrease in particle size was not obtained using PEO as a water-soluble polymer when the rotation speed was faster than 500 rpm. We did not consider the relationship between the beaker’s volume and the number of beads in this study. It was referenced previously in another study, such as used a beads-milling apparatus [7]. It might have their optimal ratio during grinding in this technic. We should consider that detail in our future study.

**Figure 5:**
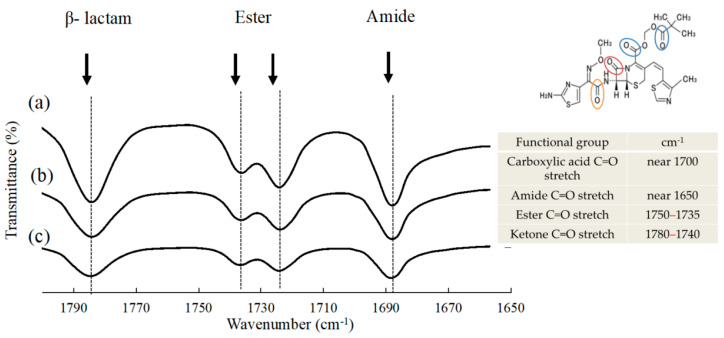
FTIR spectra of CDTR-PI crystals, NPs, and physical mixture consist of CDTR-PI, HPC-SSL, and SLS. (**a**) NPs consisting of CDTR-PI/HPC-SSL/SLS; (**b**) CDTR-PI/HPC-SSL/SLS at 40/5/1 (*w/w/w*); and (**c**) CDTR-PI crystals.

FTIR spectra of CDTR-PI crystals, NPs, and a physical mixture consisting of CDTR-PI, HPC-SSL, and SLS are shown in Figure 5. The prominent peaks observed in CDTR-PI are as follows: 1780 to ~ 1740 cm^−1^ for the stretching vibration of C=O in b-lactam, 1750 to ~1735 cm^−1^ for the stretching vibration of C=O in ester, near 1650 cm^−1^ for the stretching vibration of C=O in amide, and near 1700 cm^−1^ for the stretching vibration of C=O [23]. In this case, and as mentioned above, the hydrophilic layer composed of hydrophilic polymer chains and SLS, was considered to surround the CDTR-PI molecule; however, they did not have any interaction between each molecule in the suspension of NPs in their FTIR spectra which was focused each characteristic absorption bands (Figure 5). Additionally, there was no interaction between each polymer and SLS (data not shown). The same results were obtained for all samples of other polymers (data not shown). Furthermore, CDTR-PI and polymers with no electrical charge were used with SLS, one of the anionic surfactants in this study. In other words, it was considered that the zeta potential of NPs was shown due to the electrical charge of SLS on the polymer layers. The zeta potential indicates the strength of the electrostatic repulsive forces between particles at their surfaces [20,21]. Some reports have demonstrated that a zeta potential value of −20 mV is sufficient for a physically stable nanosuspension [24,25]. The mean zeta potential values obtained in this study ranged from about −2 to −11 mV. It was then considered that their values are not enough to maintain stable nanosuspension without aggregation between particles after preparations. However, to indicate low zeta potential values, polymer chains that have absorbed to NPs’ surface are steric hindrances; NPs are less likely to aggregate.

Moreover, as for the surfactant concentration, several reports have noted that poorly water-soluble drugs are well-grinding when the surfactant concentration is higher than its critical micelle concentration (CMC) [21,22,23,24,25,26]. On the other hand, it was reported that preparation of nano-suspension with desired particle size and well storage stability needs to select optimal stabilizers and effective process and equipment [27]. In this study, we added SLS as a surfactant, although the CMC of SLS is 0.25% (*w/v*), so the concentration was used less than its CMC because we got the optimal ratio with HPCs in our previous study using this technic [16]. Therefore, the present data suggest that HPCs and SLS concentrations less than the CMC could use together as dispersion agents when using the current technic when CDTR-PI as APIs was ground. Li et al. 2018 demonstrated that griseofulvin’s aggregation could be prevented by adding a low concentration (0.05% *w/w*) of sodium dodecyl sulfate [18], which corresponds with our results as one of the phenomena if it was considered without the grinding process and equipment. It was thought that the electrostatic repulsion works between particles in water; then, they do not aggregation if surfactant is used as a stabilizer to wet-milling methods with no electrical charge polymers, even if their electric charge is under −20 mV. We are currently investigating the effect of surfactant concentration on preventing other APIs’ aggregation and will publish more in the future.

Furthermore, as the medium’s viscosity naturally seems to affect the grinding, each sample’s viscosity was measured. As shown in Table 1, the viscosity increased when 1% of polymer concentration was used at PEO, HPC-L, and HPMC, then, the particle sizes of NPs were more reduced than the other concentrations. This suggests that the movement and collision of the beads were restricted in more viscous solutions.

**Figure 6:**
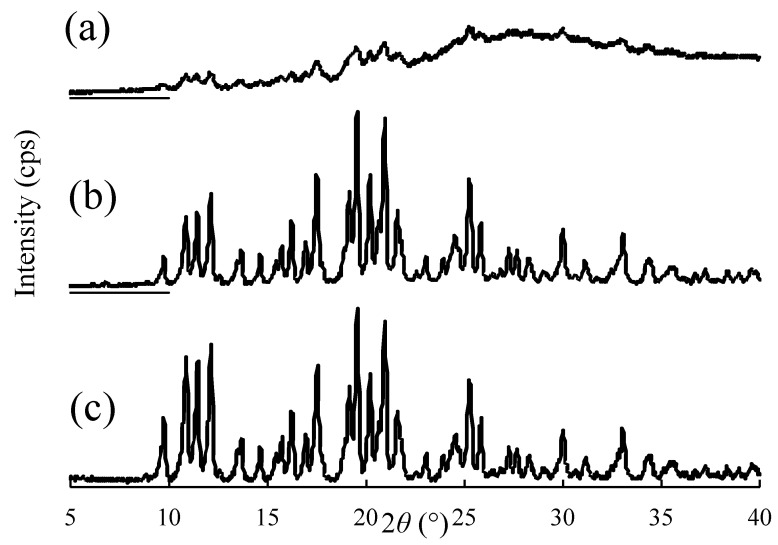
Powder X-ray diffraction patterns of CDTR-PI crystals, submicron particles, and physical mixture. (**a**) Submicron particles consisting of CDTR–PI/HPC–SSL/sodium dodecyl sulfate at 40/5/1 (*w/w/w*); (**b**) physical mixture of cefditoren pivoxil/hydroxypropyl cellulose-SSL/sodium dodecyl sulfate at 40/5/1 (*w/w/w*); and (**c**) cefditoren pivoxil crystals.

Typical examples of the PXRD patterns of CDTR-PI crystals, physical mixture, and NPs obtained in this study are shown in Figure 6. In the PXRD patterns for CDTR-PI crystals and the physical mixture, the typical diffraction peaks due to CDTR-PI crystals were observed at 2θ = 9.8°, 10.9°, 11.5°, 19.6°, 21.0°, and so on. After pulverizing, a decrease in these peaks’ diffraction intensities was observed, then, it was suggested that the crystallinity of CDTR-PI was decreased (Figure 6a). Unfortunately, it could not be distinguished between amorphous and crystalline parts in NPs, because they cannot measure thermal analysis using differential scanning calorimetry, because CDTR-PI decomposes at a melting point. Karavas et al. 2007 demonstrated that Raman mapping allows observation of the size and spatial distribution of domains where the drug existed in molecular form or is nano-dispersed [26]. This is also a subject for future investigation.

Moreover, the nanosuspension’s dispersion stability using a 0.5% (*w/v*) HPC-SSL solution, which was shown to be the smallest particle size in all samples, was evaluated by a dispersion stability analyzer demonstrated in the next section.

### 3.3. Evaluation of Dispersion Stability

The sample solutions’ dispersion stability was evaluated by a Turbiscan MA 2000 instrument, which consists of a detection head composed of a pulsed near-infrared light source, a transmission detector, and a backscattering detector, which moves vertically along a sample tube [28]. The Turbiscan has been used to evaluate emulsions’ dispersion stability and concentrated colloidal dispersions [29].

**Figure 7:**
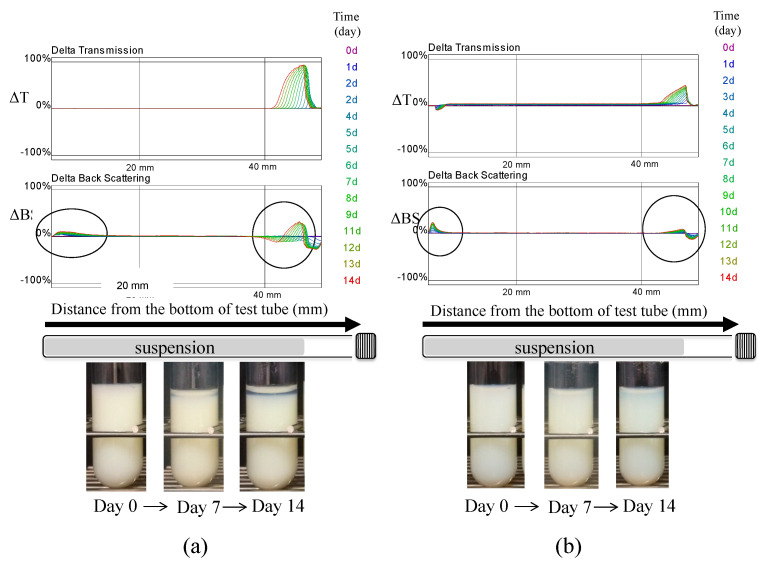
Transmission and backscattering patterns of the different suspensions using Turbiscan. (**a**) dispersion media: 0.5% HPC–SSL; (**b**) dispersion media: 0.5% HPC–SSL and sodium lauryl sulfate.

**Figure 8:**
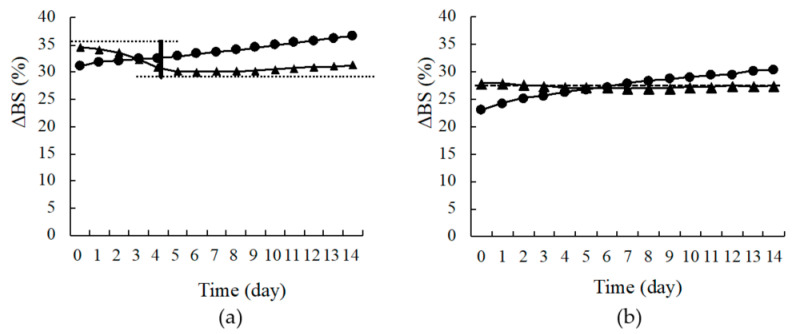
Changes in the backscattering (BS) values of various samples in the upper and lower parts. ▲: upper, ●: lower. Dispersion media: (**a**) Hydroxypropyl cellulose, 0.5% SSL; (**b**) dispersion media: hydroxypropyl cellulose, 0.5% SSL, and sodium lauryl sulfate.

In the present study, samples were evaluated by scanning every 24 h, and the percentages of transmitted light (ΔT) and backscattered light (ΔBS) were used to assess dispersion stability (Figures S2 and S3). Figure 7 shows the transmittance and backscattering patterns of nano-suspensions prepared with only 0.5% (*w/v*) HPC-SSL as reference (Figure 7a), and 0.5% (*w/v*) HPC-SSL with 0.1% (*w/v*) SLS (Figure 7b). The ΔT at the top portion of the sample tube increased with time in each sample. Besides, the values for ΔBS at the bottom and top of the sample tube increased with time. These results indicate that the particles precipitated to the bottom of the sample tube over time. However, the precipitation of particles was restricted to the NPs prepared with 0.5% (*w/v*) HPC-SSL and 0.1% (*w/v*) SLS, compared with the particles prepared with 0.5% (*w/v*) only HPC-SSL. Furthermore, the increase in ΔBS was observed at the bottom over a wide range, and a larger increase of ΔT and ΔBS values was observed at the top of the sample (Figure 7a). Changes in ΔBS were also observed at the top and the bottom of the samples (Figure 8). Although the change was small in 0.5% (*w/v*) HPC-SSL, including SLS, ΔBS decreased at the bottom of the sample over time (Figure 8a,b). In other words, it indicates that the dispersion of the nanosuspension of 0.5% (*w/v*) HPC-SSL, including SLS was stable. Moreover, it was suggested that electrostatic repulsion works between particles in water, then aggregation does not occur if anionic surfactant is used as a stabilizer to wet-milling methods with no electrical charge polymers electric charge is under −20 mV as it was mentioned above. From these results, the dispersion medium consists of 0.5% (*w/v*) HPC-SSL and 0.1% (*w/v*) SLS is applicable in this study.

### 3.4. Measurement of Solubility

The solubility of CDTR-PI crystals in water at room temperature was 2.47 μg/mL. On the other hand, the solubility of CDTR-PI in suspensions whose polymers are HPLC-SSL 0.5% and PVP 0.5% showed 8.9 mg/mL and 8.8 mg/mL, respectively. For the dissolution behaviors of CDTR-PI from CDTR-PI crystals, physical mixture, and nanosuspension, as for CDTR-PI crystals or a physical mixture, most of CDTR-PI did not dissolve in purified water. The drug release rate of CDTR-PI from a nanosuspension was 95.6%, which was noticeable at 1 min just after the start of dissolution testing (data not shown). The improvement of the solubility in the suspension observed in this study seems to be due to improved surface area of CDTR-PI crystals by grinding with stabilizers or the partial amorphization of CDTR-PI crystals by wet-milling. If the solubility can be improved, the amount of dose can be reduced. Therefore, an investigation of the nanosuspensions’ membrane permeability using cell lines such as Caco-2 cells should be carried out in the next step of this study.

## 4. Conclusions

NPs of CDTR-PI were prepared using a wet-milling method with a propeller-type stirrer and zirconia beads. NPs can be prepared using this technique with various water-soluble polymers and SLS. Although NPs in the suspension retained crystalline characteristics, these characteristics diminished with time, as determined by PXRD. The CDTR-PI, water-soluble polymers, and SLS did not interact in the solution. Besides, the solubility of CDTR-PI in the suspension improved, and the suspension had good dispersion stability. The use of SLS at a concentration less than the CMC was effective in producing the nanosuspension. Thus, we suggested a wet-milling method using a propeller-type stirrer is possible to be used in a hospital pharmacy to create nanosuspensions of poorly water-soluble drugs without the need for specialized or expensive machinery. However, the nanosuspension drug concentration in this study was small compared with the usual dose; in nanosuspension, it shows that the dosage at one time increases. On the other hand, since the solubility of CDTR-PI dramatically increased, improvement of bioavailability might be expected, it can reduce the dose. Therefore, an investigation of the nanosuspensions’ membrane permeability using cell lines such as Caco-2 cells should be carried out in the next step of this study. Raza et al. demonstrated that solid nanoparticles could be used as carriers for antimicrobial drugs and improve their bioavailability [30]. Furthermore, Venugopalarao et al. prepared the hydrogel tablets containing CDTR-PI, the release of CDTR-PI could be controlled by their formulation. Additionally, in this study, the improvement of bioavailability of CDTR-PI is expected [31].

This study proposed a simple and straightforward methodology of a wet-milling method to prepare hospital formulation in this study. Unfortunately, it took 24 h to obtain the NPs, so finding more efficient conditions, including qualitative or quantitative pharmaceutical parameters and drug concentration, should be a subject of future investigation. Besides, we should consider the suspension’s storage stability in each condition if the suspension is applied to hospital formulations. We have already reported that this technique applies to Rebamipide, one of the poorly water-soluble APIs [13]. Henceforward, also for the other poorly water-soluble APIs, we would like to investigate whether this technique can apply to prepare the nano-suspensions as the hospital formulations.

## Figures and Tables

**Table 1:** Particle diameter and zeta potential of ground particles of CDTR-PI indifferent dispersion media.

Dispersing Media			Particle Size			Zeta Potential (mV) ± S.D.	Viscosity (mPa·s)
Polymer		Surfactant	Mean (nm) ± S.D.	D_50_ (nm) ± S.D.	PDI		
PEO	0.25%	SLS 0.1%	167.6 ± 2.8	152.1 ± 5.5	0.227	−0.7 ± 0.4	1.31
	0.5%		160.4 ±4.0	301.0 ± 22.0	0.171	−1.4 ± 0.4	1.71
	1.0%		204.7 ± 3.9	187.7 ± 2.4	0.229	0.3 ± 0.8	2.91
HPC-L	0.25%		142.8 ± 1.4	140.5 ± 4.1	0.188	−7.5 ± 1.4	1.29
	0.5%		133.6 ± 0.6	127.8 ± 2.9	0.172	−11.5 ± 2.4	1.96
	1.0%		208.1 ± 1.2	205.9 ± 1.2	0.170	−4.6 ± 0.6	3.76
HPC-SL	0.25%		137.0 ± 0.3	137.8 ± 7.5	0.182	−4.9 ± 1.2	1.18
	0.5%		123.3 ± 1.9	121.5 ± 7.3	0.176	−6.1 ± 1.2	1.50
	1.0%		158.8 ± 1.3	152.7 ± 1.4	0.135	−2.4 ± 0.7	2.41
HPC-SSL	0.25%		128.7 ± 0.4	128.2 ± 6.3	0.176	−9.8 ± 1.1	1.07
	0.5%		116.1 ± 0.4	111.7 ± 5.2	0.167	−3.7 ± 0.4	1.21
	1.0%		135.3 ± 9.0	137.5 ± 14.0	0.221	−4.9 ± 0.3	1.68
HPMC	0.25%		209.6 ± 2.0	212.0 ± 2.9	0.226	−3.5 ± 0.8	1.72
	0.5%		275.1 ± 4.3	273.7 ± 12.1	0.221	−0.8 ± 0.3	2.95
	1.0%		413.8 ± 1.0	386.4 ±3.8	0.182	−2.9 ± 0.7	7.30
PVP	0.25%		134.0 ± 2.2	134.4 ± 9.0	0.187	−7.2 ± 2.6	1.00
	0.5%		173.2 ± 3.0	175.6 ± 10.7	0.219	−5.5 ± 0.5	1.05
	1.0%		127.4 ± 0.4	120.5 ± 6.3	0.158	−4.9 ± 0.2	1.15

Data represent the mean ± S.D. of three experiments. Mean is the mean diameter based on cumulus fitting analysis. D_50_ is the diameter at 50% of the cumulative volume distribution.

## Data Availability

The data in the form of excel sheets presented in this study are available on request from the corresponding author.

## Supplementary Materials

The following are available online at https://www.mdpi.com/article/10.3390/pharmaceutics13040482/s1, Figure S1: Chemical structure of cefditoren pivoxil. Figure S2: Schematic diagram of Tubiscan. Figure S3: Principles behind the measurement of sedimentation behavior using Turbiscan. (a) Change in Δ transmission (%) and (b) change in Δ backscattering (%) before and after dispersion. Table S1: The results of performed statistical analysis by Tukey-Kramer test to particle size. Table S2: The particle size and zeta potential of ground particles of CDTR-PI using various dispersing media.
