# Supplementary material for: Testing a Benchtop Wet-Milling Method for Preparing Nanoparticles and Suspensions as Hospital Formulations
Source: Pharmaceutics. 2021 Apr 2;13(4):482. doi: 10.3390/pharmaceutics13040482 (PMC8065928; doi:10.3390/pharmaceutics13040482)
Supplement: Supplementary file 1 [file pharmaceutics-13-00482-s001.pdf]

pharmaceutics

Supplementary Materials: Testing a Benchtop Wet-Milling Method for Preparing Nanoparticles and Suspensions as Hospital Formulations

Yayoi Kawano, Yuichiro Shimizu and Takehisa Hanawa *

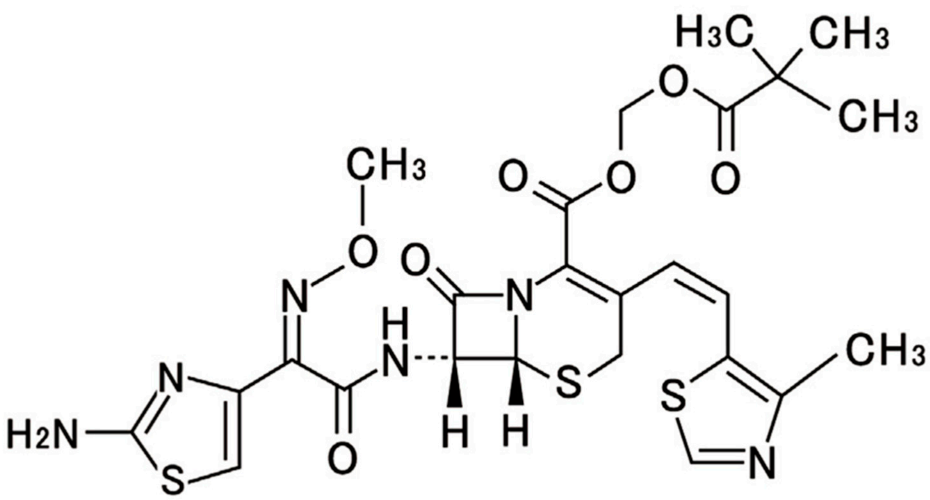

Figure S1. Chemical structure of cefditoren pivoxil.

Publisher's Note: MDPI stays neutral with regard to jurisdictional claims in published maps and institutional affiliations.

Copyright: © 2021 by the authors. Submitted for possible open access publication under the terms and conditions of the Creative Commons Attribution (CC BY) license (http://creativecommons.org/licenses/by/4.0/).

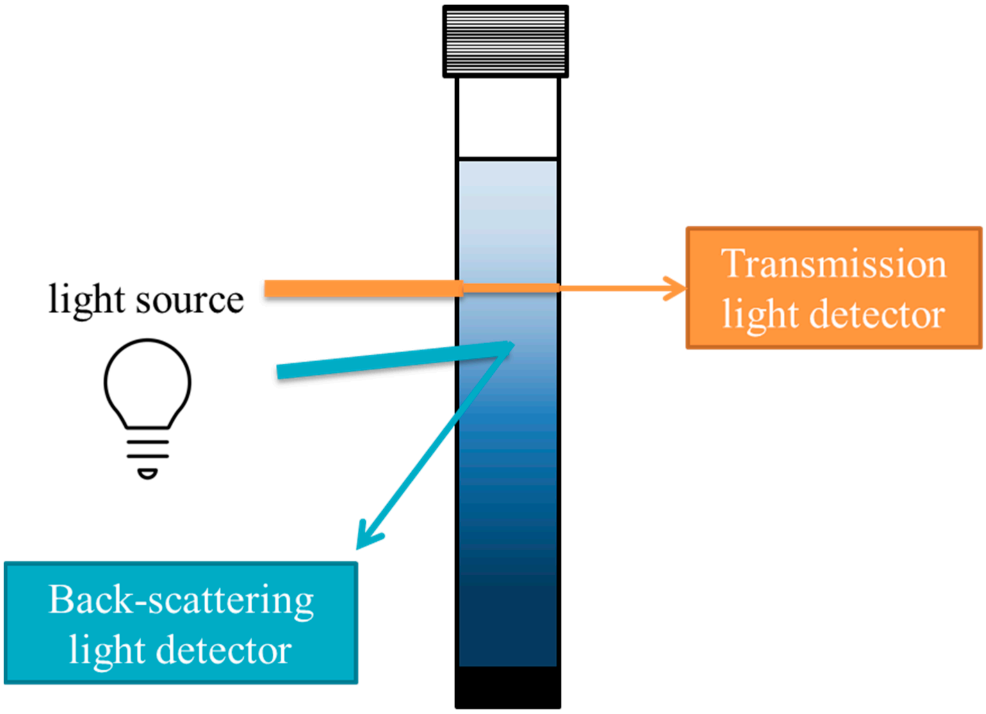

Figure S2. Schematic diagram of Tubiscan.

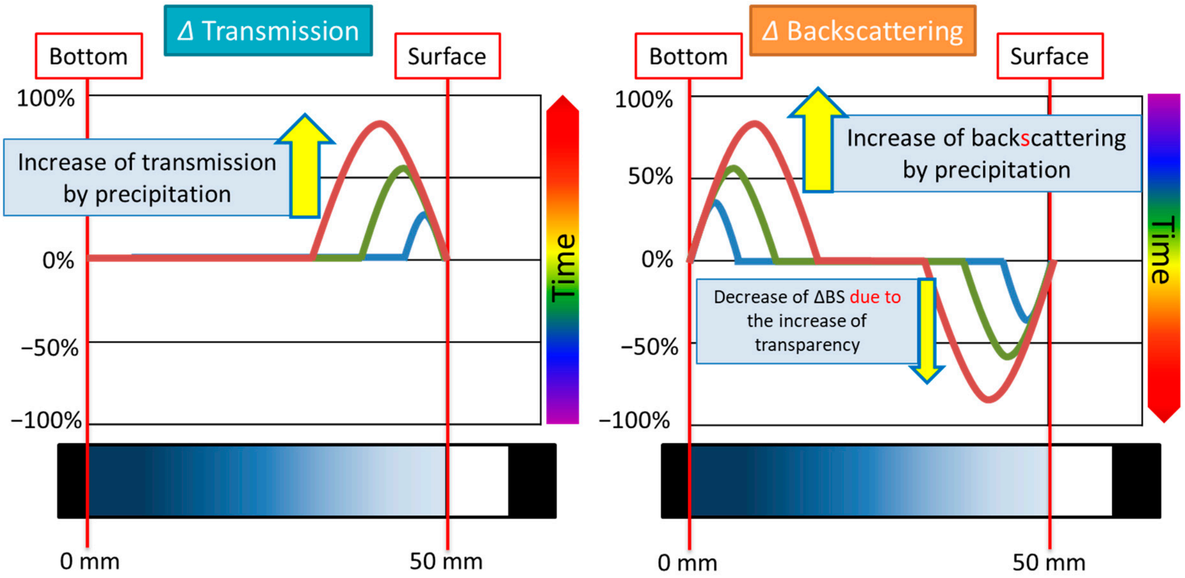

(a)

Figure S3. Principles behind the measurement of sedimentation behavior using Turbiscan. (a) Change in *Δ* transmission (%) and (b) change in *Δ* backscattering (%) before and after dispersion.

Table S1. The results of performed statistical analysis by Tukey-Kramer test to particle size

		**PEO**			**HPC-L**			**HPC-SL**			**HPC-SSL**			HPMC			PVP		
		0.25%	0.5%	1.0%	0.25%	0.5%	1.0%	0.25%	0.5%	1.0%	0.25%	0.5%	1.0%	0.25%	0.5%	1.0%	0.25%	0.5%	1.0%
PEO	0.25%			**	**	**	**	**	**		**	**	**	**	**	**	**	**	**
	0.5%			**	**	**	**	**	**		**	**	**	**	**	**	**	**	**
	1.0%	**	**		**	**		**	**	**	**	**	**	**		**	**	**	**
HPC-L	0.25%	**	**	**		*	**		**	**	**	**		**	**	**		**	**
	0.5%	**	**	**	*		**		*	**		**		**	**	**		**	
	1.0%	**	**		**	**		**	**	**	**	**	**		**	**	**	**	**
HPC-SL	0.25%	**	**	**			**		**	**		**		**	**	**	**	**	*
	0.5%	**	**	**	**	**	**	**		**			**	**	**	**	**		
	1.0%			**	**	**	**	**	**		**	**	**	**	**	**	**	**	**
HPC-SSL	0.25%	**	**	**	**		**			**		**		**	**	**			
	0.5%	**	**	**	**	**	**	**		**	**		**	**	**	**	**		**
	1.0%	**	**	**			**		**	**		**		**	**	**		**	
HPMC	0.25%	**	**	**	**	**		**	**	**	**	**	**		**	**	**	**	**
	0.5%	**	**		**	**	**	**	**	**	**	**	**	**		**	**	**	**
	1.0%	**	**	**	**	**	**	**	**	**	**	**	**	**	**		**	**	**
PVP	0.25%	**	**	**			**	**	**	**		**		**	**	**		*	
	0.5%	**	**	**	**	**	**	**		**			**	**	**	**	*		
	1.0%	**	**	**	**		**	*		**		**		**	**	**			

*: $p < 0.05$, **: $p < 0.01$.

Table S2. The particle size and zeta potential of ground particles of CDTR-PI using various dispersing media.

**Dispersing media**		**Particle size**				**Zeta potential (mV) ±S.D.**		**Viscosity (mPa s)**
		**Mean (nm) ±S.D.**		**D50 (nm) ±S.D.**				
water	-	475.6	± 117.4	239.6	± 15.2	0.7	± 1.9	0.96
PEO	0.5%	319.7	± 20.3	271.2	± 12.1	-2.2	± 1.4	1.80
PVP	0.5%	1482.5	± 116.6	6010.7	± 227.2	-5.9	± 1.2	1.10
HPC-SSL	0.5%	508.9	± 12.4	407.8	± 11.3	-2.7	± 0.5	1.23
SLS	0.1%	210.1	± 4.6	138.0	± 1.4	-5.9	± 0.7	0.96
Tween 80	0.1%	3853.2	± 1815.3	6842.4	± 210.7	-2.8	± 0.9	0.94

Data represent mean ±S.D. of three determinations. Mean is a mean diameter based on Cumulants fitting analysis. D50 is diameter at the 50% of the cumulative volume distribution.
